# Outcomes of Revascularisation for Treating Lifestyle-Limiting Intermittent Claudication in Aboriginal and Torres Strait Islander People and Non-Indigenous Patients from North Queensland: A Retrospective Cohort Study

**DOI:** 10.3390/jcm13113339

**Published:** 2024-06-05

**Authors:** Shannon Wong, Shivshankar Thanigaimani, James Charles, Donald Whaleboat, Jonathan Golledge

**Affiliations:** 1 Queensland Research Centre for Peripheral Vascular Disease, College of Medicine and Dentistry, James Cook University, Townsville, QLD 4811, Australia; shannon.wong@my.jcu.edu.au (S.W.); shiv.thanigaimani@jcu.edu.au (S.T.); 2 First Peoples Health Unit, Griffith University, Brisbane, QLD 4222, Australia; james.charles@griffith.edu.au; 3 Anton Breinl Research Centre for Health Systems Strengthening, Australian Institute of Tropical Health and Medicine, James Cook University, Townsville, QLD 4811, Australia; donald.whaleboat@jcu.edu.au; 4 The Department of Vascular and Endovascular Surgery, Townsville University Hospital, Townsville, QLD 4814, Australia

**Keywords:** peripheral artery disease, revascularisation, intermittent claudication, amputation, Aboriginal and Torres Strait Islander health

## Abstract

**Background:** This retrospective analysis of an ongoing prospective cohort study aimed to assess the outcome of revascularisation for treating lifestyle-limiting intermittent claudication caused by peripheral artery disease (PAD) in Aboriginal and Torres Strait Islander Peoples and non-Indigenous North Queenslanders. **Methods:** Consenting patients with PAD who underwent endovascular or open revascularisation procedures for treating lifestyle-limiting intermittent claudication were included. The primary outcome measure was major adverse limb events (MALEs), defined as major amputation or the requirement for repeat open or endovascular revascularisation. **Results:** Of the 378 included patients, 18 (4.8%) identified as Aboriginal and/or Torres Strait Islander Peoples. During a mean follow-up (standard deviation) of 6.0 (3.9) years, the incidence of MALE was similar in the Aboriginal and Torres Strait Islander People and non-Indigenous Australians (absolute percentage: 50.0% vs. 40.6%, log rank *p* = 0.59). In both unadjusted and adjusted analyses, Aboriginal and Torres Strait Islander Peoples and non-Indigenous Australians had similar risks of MALE (unadjusted hazard ratio, HR, 1.20, 95% confidence interval, CI, 0.61, 2.36; adjusted HR 1.02, 95%CI 0.50, 2.06). **Conclusions:** This study suggests that Aboriginal and Torres Strait Islander People are under-represented in the population of patients undergoing revascularisation to treat intermittent claudication. Due to small numbers it cannot be reliably concluded that Aboriginal and Torres Strait Islander People and non-Indigenous Australians have similar rates of MALE.

## 1. Introduction

Peripheral artery disease (PAD) is an atherothrombotic occlusion of the arteries supplying the lower limbs. In 2015, about 6% of adults worldwide were reported to have PAD, which represents approximately 250 million people [1,2]. The prevalence has since had a significant relative increase of 22.6% in low- and middle-income countries compared to 4.5% in high-income countries [3]. The most well-recognised symptom of PAD is intermittent claudication, which limits activity and reduces health-related quality of life [4,5]. Current guidelines recommend surgical or endovascular revascularisation in patients with intermittent claudication who have failed to be successfully managed with exercise therapy and medical management [4,6].

The existing knowledge about PAD in Aboriginal and Torres Strait Islander Australians is limited. Assessment of 135 Aboriginal and Torres Strait Islander Australians with diabetes from the Royal Darwin Hospital using the ankle brachial index (ABI) identified PAD in 17 (13%) people. After adjusting for other risk factors, the prevalence of PAD in this Aboriginal and Torres Strait Islander population was reported to be 3-fold higher than in a sample of 763 mainly non-Indigenous Australians with diabetes recruited from across Australia [7]. Another study of 73 Aboriginal and Torres Strait Islander Peoples with PAD found that they were much more likely to present with chronic limb-threatening ischaemia as compared to 242 non-Indigenous patients with PAD, who were more likely to present with intermittent claudication [8]. Aboriginal and Torres Strait Islander Peoples were also found to have a greater prevalence of distal artery disease, which may explain the differences in clinical presentation between the populations [8]. Aboriginal and Torres Strait Islander Australians with PAD have also been reported to be at an approximately 5-fold greater risk of major adverse cardiovascular events [9] and to have a between 2- and 3-fold greater risk of major amputation compared to non-Indigenous Australians [10]. The higher burden of disease in Aboriginal and Torres Strait Islander Australians may be explained by the greater prevalence of risk factors for PAD and failure of current treatment algorithms to control these risk factors, including diabetes, renal insufficiency and hypertension [11,12]. The higher burden of disease is likely related to the deficiency of culturally appropriate care, causing language and cultural barriers to health literacy [13] and discouraging Aboriginal and Torres Strait Islander Australians from attending for treatment [14,15,16]. Importantly, despite the higher incidence of chronic limb-threatening ischaemia amongst Aboriginal and Torres Strait Islander peoples, the rate of lower extremity revascularisation was found to be the same as in non-Indigenous patients, which may be due to poorer access to surgery or the greater challenges of revascularisation in the presence of severe distal disease [8].

Most of the research on PAD in Aboriginal and Torres Strait Islander populations is focused on people presenting with chronic limb-threatening ischaemia. Little is known about the patients who present with non-limb-threatening ischaemia and what the outcome are of revascularisation for treating intermittent claudication. The aim of this study was to assess the outcome of revascularisation in Aboriginal and Torres Strait Islander People as compared with non-Indigenous Australians for treating lifestyle-limiting intermittent claudication. The main outcome was the incidence of major adverse limb events (MALEs). This was important to assess as a gauge of the safety of this intervention in Aboriginal and Torres Strait Islander People with intermittent claudication.

## 2. Materials and Methods

### 2.1. Study Design

This was a retrospective analysis of data from an ongoing prospective cohort study that aims to identify risk factors associated with the diagnosis and outcome of vascular disease. For inclusion in this study, participants need to present for treatment of lifestyle-limited intermittent claudication caused by PAD, having failed to improve with medical management alone and having been treated by an endovascular or open revascularisation procedure at Townsville University Hospital or Mater Hospital Townsville. Medical management aimed to address modifiable risk factors via prescription of anti-platelet and anti-coagulation drugs and medications to control low-density lipoprotein cholesterol, blood pressure, and diabetes and aid smoking cessation as per the ESVS guidelines [3]. PAD was defined as an ankle brachial index (ABI)  < 0.9 and/or imaging demonstrating >50% stenosis or occlusion of the lower limb arteries. All the patients were assessed by a Royal Australasian College of Surgeons accredited vascular surgeon who diagnosed intermittent claudication based on a history of cramping calf pain on walking and clinical evidence of PAD. All the surgeons reserved revascularisation for patients with lifestyle-limiting intermittent claudication persisting after a trial of medical management. Patients with other presentations of PAD, such as chronic limb-threatening ischaemia, were excluded.

This research study aligns with the Indigenous Research Excellence criteria outlined by the National Health and Medical Research Council [17]. Our prior consultations with Aboriginal and Torres Strait Islander Peoples and health professionals indicated that closing the gap in the health outcomes of PAD and creating sustainable changes in evidence-based practice and health policy for diagnosing and treating PAD in this population are key concerns [18,19]. All the participants provided informed written consent and the study was approved by the Townsville Hospital and Health Service Ethics Committee (HREC/14/QTHS/203). This study was reported according to the Strengthening the Reporting of Observational Studies in Epidemiology (STROBE) checklist (Table A1) [20].

### 2.2. Aboriginal and Torres Strait Islander Ethnicity

Aboriginal and Torres Strait Islander ethnicity was recorded based on self-identification by the patients at the time of recruitment. This data were extracted from hospital medical records.

### 2.3. Risk Factors

Demographic and clinical risk factors were all recorded at the time of the participants’ first presentation to the outpatient clinic and collected from interviews and medical records, as previously reported [21]. The collected variables included age, sex, smoking history, diabetes, hypertension, ischaemic heart disease, stroke, end-stage renal failure, prior peripheral revascularisation (endovascular or open surgical revascularisation), and current medications.

Smoking was classified as current smoking (smoked within the last month), former smoking (ceased smoking for more than 1 month) and never smoking according to the participants’ self-reporting [21]. Hypertension was defined as a documented history of past diagnosis or anti-hypertensive treatment [21]. Diabetes was defined as a documented fasting blood glucose concentration ≥ 7.0 mM or HbA1c ≥ 6.5%, or a history of treatment for diabetes [21]. Ischaemic heart disease was defined by a history of angina, myocardial infarction, or past treatment of ischaemic heart disease [21]. Stroke was defined as a documented episode of an acute focal neurological deficit of more than 24 h duration with evidence of cerebral infarction on a brain computed tomography (CT) scan or magnetic resonance imaging (MRI) [22]. End-stage renal failure was defined as requiring dialysis [23]. All the prescribed medications, including anti-thrombotic medications (including aspirin, clopidogrel, other antiplatelets or warfarin), anti-hypertensive medications (including angiotensin-converting enzyme inhibitors, angiotensin receptor blockers, calcium channel blockers, beta blockers or other blood pressure-lowering drugs), diabetes medications (including metformin, insulin, and other glucose-lowering medications), and statins, were collected.

### 2.4. Outcome Assessment

Outcome data were collected at follow-up appointments and subsequently checked by thorough review of medical records. The primary outcome was MALEs, which were defined as a major amputation (above or below knee or hindquarter) or the requirement for repeat open or endovascular peripheral limb revascularisation. The secondary outcome was the requirement for repeat revascularisation alone. The time to a MALE and repeat revascularisation were defined as the time in years from the participants’ index revascularisation procedure to the first event.

Patients were followed up at outpatient appointments and/or as an inpatient as part of their normal medical care. Participants were offered a review appointment 6 months after their initial assessment, and then yearly unless their symptoms or imaging findings changed [24]. Follow-up was defined as the time in years from the patients’ index peripheral revascularisation procedure and concluded at the date of the last inpatient or outpatient review or at the participant’s death.

### 2.5. Sample Size

The required sample size was estimated according to the planned Cox regression analysis focused on MALEs. Based on prior studies, the incidence of MALEs was estimated to be about 30–40% over a minimum of 2 years [25,26]. The regression analysis was planned to include up to 10 variables, including age, sex, smoking, diabetes, hypertension, ischaemic heart disease, stroke, end-stage renal failure, previous history of revascularisation (endovascular or open surgical revascularisation), and anti-thrombotic medications. Monte Carlo simulations suggested that a multivariable regression model is sufficiently powered when 10 outcome events per degree of freedom of the predictor variables are observed [27]. Based on these estimates, a sample size of approximately 300 patients with at least 2 years of follow-up was adequate.

### 2.6. Data Analysis

Independent samples *t*-tests and chi-square tests were used to assess the differences between Aboriginal and Torres Strait Islander Peoples and non-Indigenous participants at entry. The time to a MALE and the time to repeat revascularisation were estimated using Kaplan–Meier probability curves and the log rank test was used to determine if there was a significant difference between the groups. Cox proportional hazards regression modelling was performed to investigate the association between Aboriginal and Torres Strait Islander ethnicity and MALEs or repeat revascularisation. The regression models were adjusted for potentially confounding demographic and clinical risk factors, including those found to be significantly different (*p* < 0.05) between comparator groups and other known clinically relevant risk factors (including age, diabetes, ischaemic heart disease, current smoking, and anti-thrombotic use) [9]. Schoenfeld’s test was performed to ensure that the proportional hazard assumptions were met for the multivariable models. Hazard ratios with a 95% confidence interval were reported for each variable included in the final model.

To select the best-fitting regression model, multiple statistical tests were conducted. ANOVA was performed to determine if there was any significant difference between the models. The Akaike information criterion (AIC) was calculated for all the models, which is a measure of model fitness that penalises any addition of unnecessary covariates. Lower values indicate a better-fitting model and a model with an AIC difference (delta-AIC) of more than 2 is considered significantly better than the model it is being compared to [28]. A parsimonious model was selected as the one with the lowest AIC value [28]. The concordance index (C-index) was calculated as a measure of the discriminatory power (performance) of the model, meaning how well the model can ascertain and correctly rank the relative risk of the individuals in relation to their respective follow-up period [29]. We also evaluated the validity of the model using the likelihood ratio test, which indicates whether the multivariable model significantly improved the fitness compared to the reduced model (based on population only, without covariates) [30]. Identification of the confounding variables used for the adjusted analysis and selection of the best-fitting Cox proportional hazard regression model are detailed in Appendix B.

All the analyses were performed using IBM SPSS Statistics, Version 27.0 (IBM Corp. Armonk, NY, USA) and R statistical program version 4.3.1. A *p* value ≤ 0.05 indicated statistical significance.

## 3. Results

### 3.1. Baseline Demographics

**Figure 1:**
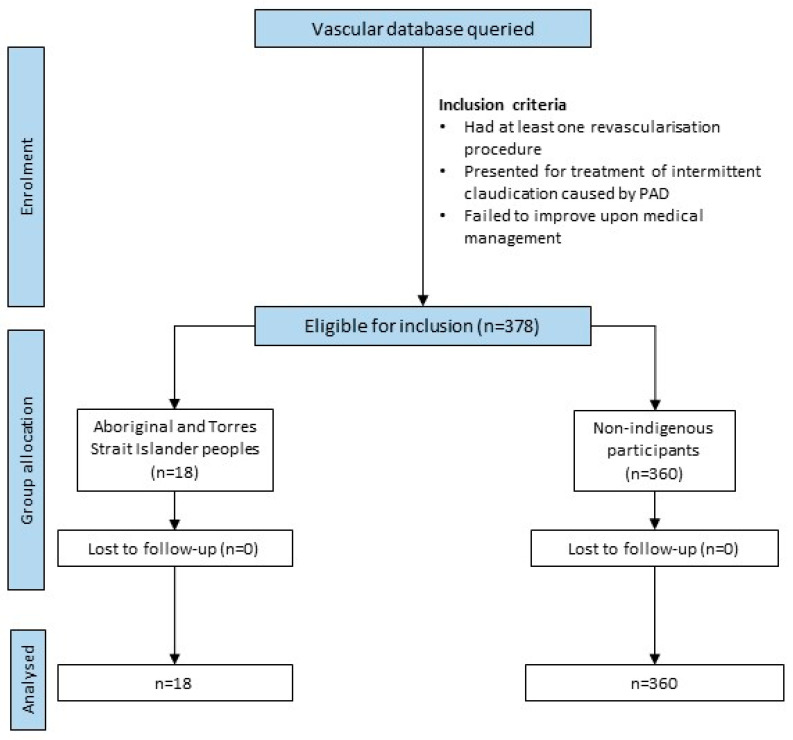
Flowchart for the retrospective cohort data collection and analysis.

A total of 378 patients were included, of whom 18 (4.8%) identified as being Aboriginal and Torres Strait Islander Peoples (Table 1, Figure 1). The Aboriginal and Torres Strait Islander Peoples were significantly younger and more likely to have diabetes and be prescribed diabetes medications than the non-Indigenous participants (Table 1). There was no significant difference in the sex distribution, type of index revascularisation, site of intervention, incidence of major amputation, number of reinterventions, or duration of follow-up between the cohorts. There was also no significant difference between the groups regarding other risk factors for PAD progression, including smoking, previous revascularisation procedures, hypertension, ischaemic heart disease and other prescribed medications.

### 3.2. Association between Aboriginal and Torres Strait Islander Ethnicity and MALEs

**Figure 2:**
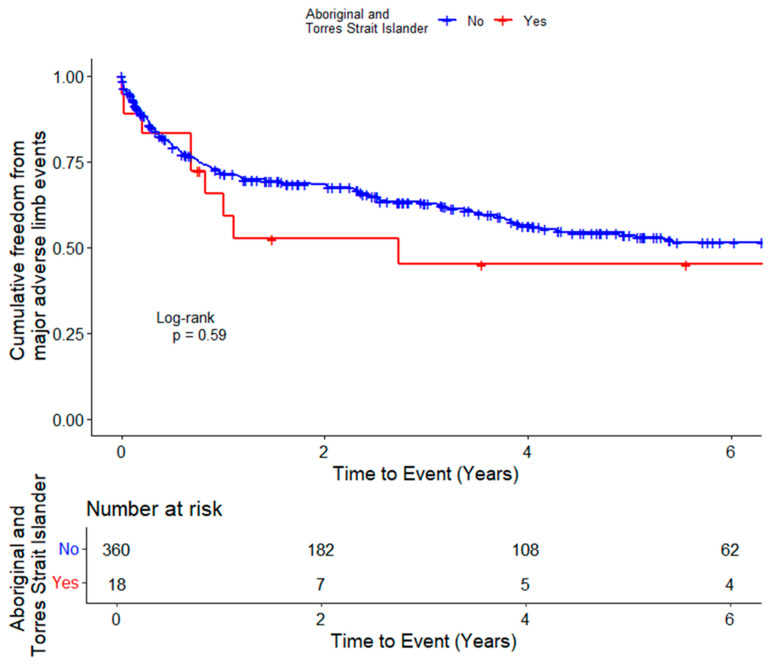
Kaplan–Meier curve showing the cumulative probability of major adverse limb events in Aboriginal and Torres Strait Islander Peoples and non-Indigenous Australians with intermittent claudication who have had revascularisation.

**Figure 3:**
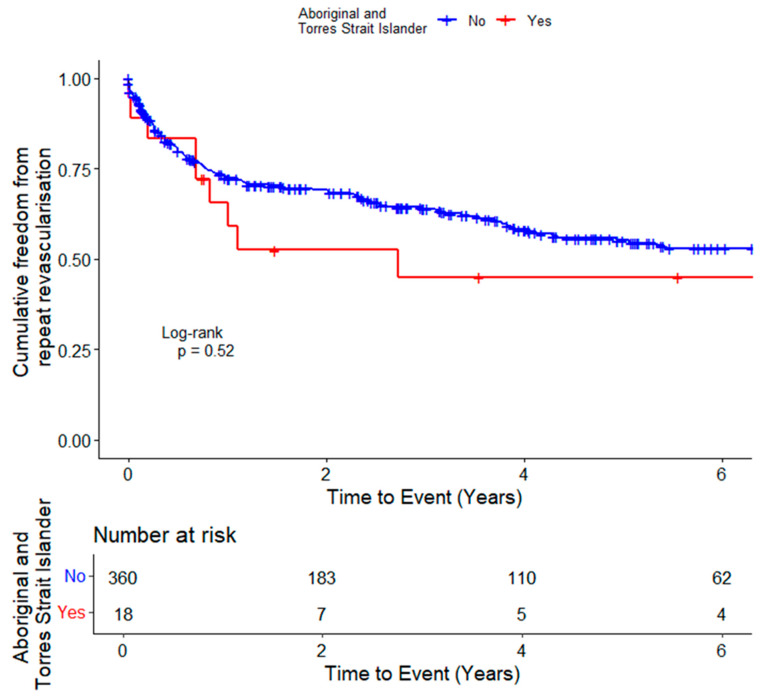
Kaplan–Meier curve showing the cumulative probability of repeat revascularisation alone in Aboriginal and Torres Strait Islander Peoples and non-Indigenous participants with intermittent claudication.

Participants were followed for a mean (SD) of 6.0 (3.9) years. The incidence of MALEs (log rank test, *p* = 0.59) and repeat revascularisation alone (log rank test, *p* = 0.52) were not significantly different in the Aboriginal and Torres Strait Islander Peoples and non-Indigenous participants (Figure 2 and Figure 3).

In the unadjusted Cox proportional hazard analyses, Aboriginal and Torres Strait Islander Peoples did not have a significantly higher risk of MALEs (HR 1.20, 95% CI 0.61–2.36, *p* = 0.595) or repeat revascularisation alone (HR 1.25, 95% CI 0.64–2.45, *p* = 0.522) than non-Indigenous participants (Table 2). In the analysis adjusted for age, diabetes and ischaemic heart disease, Aboriginal and Torres Strait Islander People had a similar risk of MALEs (HR 1.02, 95% CI 0.50–2.06, *p* = 0.964) or repeat revascularisation alone (HR 1.05, 95% CI 0.52–2.13, *p* = 0.893) as non-Indigenous participants.

## 4. Discussion

To the best of our knowledge, this is the first study of Aboriginal and Torres Strait Islander Peoples with intermittent claudication and their outcomes after revascularisation. Only 5% of the participants undergoing revascularisation for intermittent claudication were Aboriginal and Torres Strait Islander Peoples. In contrast, previously published series from the same geographical region studying patients presenting with chronic limb-threatening ischaemia found that 25% were Aboriginal and Torres Strait Islander Peoples [8]. This suggests that Aboriginal and Torres Strait Islander Peoples are under-represented in this population having revascularisation to treat intermittent claudication. The reasons for this are unclear but could include different disease presentation, under diagnosis or reduced access to treatment. Previous studies have suggested important contributors to delayed presentation of Aboriginal and Torres Strait Islander Peoples to include financial barriers, other competing health priorities, mistrust of the health system and lack of culturally appropriate services [8,14,15,31,32,33]. The findings of this study suggest that early detection of PAD in Aboriginal and Torres Strait Islander Peoples could provide an opportunity for medical management to reduce the risk of progression to chronic limb-threatening ischaemia and lower the risk of other cardiovascular events.

Due to the small sample size, we could not reliably conclude that Aboriginal and Torres Strait Islander Peoples with intermittent claudication undergoing revascularisation were no more likely than non-Indigenous participants to have a MALE. However, this should be taken in context with multiple previous studies with larger sample sizes reporting higher rates of major amputation and major adverse cardiovascular events in Aboriginal and Torres Strait Islander Peoples with PAD than in non-Indigenous people [8,9,10]. This may reflect the better outcomes if PAD can be identified at an earlier stage, further supporting the need for early detection of PAD in Aboriginal and Torres Strait Islander Peoples. These findings may also reflect the clinical environment at the Townsville health services and the approach of staff toward Aboriginal and Torres Strait Islander Peoples in their care. Overall, this study suggests that Aboriginal and Torres Strait Islander Peoples with PAD-related walking impairment who have failed conservative management have similar results of revascularisation as non-Indigenous Australians.

This study had several strengths and limitations. There was a long duration of follow-up, which allowed us to reliably estimate the long-term outcomes. Also, the use of multiple statistical tests to select the best-fitting multivariate regression model allowed us to adjust for variables that may have confounded our results. The limitations included the inadequately powered small population of self-identified Aboriginal and Torres Strait Islander Peoples, highlighting one of the study’s main findings that intermittent claudication is under-diagnosed in this population. Although statistical analysis suggested no difference, there is a significant risk of a type-II error for the outcomes measured and heterogeneity in the type of interventions offered to specific groups. Secondly, the retrospective analysis may have conferred selection bias as the study participants may not have been representative of the target population, although the inclusion criteria were determined a priori to mitigate potential bias. Thirdly, the population was limited to North Queensland and may not be generalisable to other Aboriginal and Torres Strait Islander Peoples. Fourthly, no formal exercise programs for peripheral artery disease were available as these are not publicly funded in Australia. Lastly, the definition of a MALE used in the current study did not address the suggested criteria, which involved new diagnosis of acute or chronic limb threatening ischaemia, identification of new stenosis or occlusion after previous intervention. However, due to the retrospective nature of the study, a clear redefinition was needed for the transparency and reproducibility of the results.

Further studies are needed to elucidate the reasons for the under-representation of Aboriginal and Torres Strait Islander Peoples amongst the population presenting with intermittent claudication and culturally appropriate ways of early diagnosis and treatment of PAD in this population.

## 5. Conclusions

This study found that Aboriginal and Torres Strait Islander Peoples are under-represented in the population of patients undergoing revascularisation to treat intermittent claudication. Therefore, it cannot be reliably concluded that Aboriginal and Torres Strait Islander Peoples and non-Indigenous Australians have similar rates of MALEs following treatment.

## Figures and Tables

**Table 1:** Baseline characteristics of the included participants.

Variables	Total Population (*n* = 378)	Non-Indigenous Participants (*n* = 360)	Aboriginal and Torres Strait Islander Peoples (*n* = 18)	χ^2^ or *t*	*p*
Demographic Variables					
Age (years)	64.6 ± 9.3	65.0 ± 9.1	56.9 ± 8.9	3.688	<0.001
Male	289 (76.5)	278 (77.2)	11 (61.1)	2.472	0.15
Smoking				0.742	0.69
- Current smoker	152 (40.2)	144 (40.0)	8 (44.4)		
- Former smoker	199 (52.6)	191 (53.1)	8 (44.4)		
- Never smoker	27 (7.1)	25 (6.9)	2 (11.2)		
Procedure Data					
Type of revascularisation				1.646	0.95
- Endovascular	198 (52.4)	186 (51.7)	12 (66.7)		
Plain balloon angioplasty	78 (20.6)	73 (20.3)	5 (27.8)		
Stent	118 (31.2)	113 (31.4)	5 (27.8)		
Atherectomy	2 (0.5)	0 (0.0)	2 (11.1)		
- Open	176 (46.6)	170 (47.2)	6 (33.3)		
Bypass	104 (27.5)	98 (27.2)	5 (27.8)		
Endarterectomy alone	53 (14.0)	52 (14.4)	1 (5.6)		
Bypass and endarterectomy	19 (5.0)	19 (5.3)	0 (0.0)		
- Hybrid	2 (0.5)	2 (0.6)	0 (0.0)		
- Missing data	2 (0.5)	2 (0.6)	0 (0.0)		
Site of intervention				13.889	0.09
- Aortoiliac	96 (25.4)	92 (25.6)	4 (22.2)		
- Femoropopliteal	92 (24.3)	89 (24.7)	3 (16.7)		
- Superficial femoral	54 (14.2)	50 (13.9)	4 (22.2)		
- Popliteal	5 (1.3)	4 (1.1)	1 (5.6)		
- Tibial	2 (0.5)	2 (0.6)	0 (0.0)		
- Peroneal	0 (0.0)	0 (0.0)	0 (0.0)		
- Other	14 (3.7)	11 (3.1)	3 (16.7)		
- Multiple sites	108 (28.6)	105 (29.2)	3 (16.7)		
- Missing data	7 (1.9)	7 (1.9)	0 (0.0)		
Major amputation	19 (5.0)	18 (5.0)	1 (5.6)	1.833	0.18
- Below knee	8 (2.1)	7 (1.9)	1 (5.6)		
- Above knee	14 (3.7)	14 (3.9)	0 (0.0)		
Number of reinterventions	1.0 ± 1.9	1.1 ± 1.9	0.8 ± 1.0	0.484	0.63
Duration of follow-up (years)	6.0 ± 3.9	6.0 ± 3.9	6.7 ± 4.4	−0.704	0.49
Medical History					
Previous peripheral endovascular revascularisation	85 (22.5)	81 (22.5)	4 (22.2)	0.001	1.00
Previous peripheral open revascularisation	46 (12.2)	43 (11.9)	3 (16.7)	0.358	0.47
Hypertension	282 (74.6)	269 (74.7)	13 (72.2)	0.057	0.79
Diabetes	125 (33.1)	113 (31.4)	12 (66.7)	9.639	0.004
Ischaemic heart disease	181 (47.9)	171 (47.5)	10 (55.6)	0.446	0.63
Stroke	29 (7.7)	28 (7.8)	1 (5.6)	0.120	0.73
End-stage renal failure	2 (0.5)	1 (0.3)	1 (5.6)	9.073	0.09
Medication History					
Anti-thrombotic	319 (84.4)	303 (84.2)	16 (88.9)	0.290	0.59
Anti-hypertensive	282 (74.6)	268 (74.4)	14 (77.8)	0.101	0.75
Statin	282 (74.6)	268 (74.4)	14 (77.8)	0.101	0.751
Anti-glycaemic	110 (29.1)	100 (27.8)	10 (55.6)	6.411	0.016

Data are shown as the mean (standard deviation) or number (%). The normality distribution of the continuous variables was tested using the Shapiro–Wilk test.

**Table 2:** Cox proportional hazard analyses for the association between Aboriginal and Torres Strait Islander ethnicity and major adverse limb events and repeat revascularisation alone in participants who had revascularisation to treat lifestyle-limiting intermittent claudication.

Adverse Event	Unadjusted Hazard Ratio (95% CI)	*p* Value	Adjusted Hazard Ratio (95% CI) *	*p* Value
Major adverse limb events	1.20 (0.61–2.36)	0.595	1.02 (0.50–2.06)	0.964
Repeat revascularisation	1.25 (0.64–2.45)	0.522	1.05 (0.52–2.13)	0.893

* Adjusted for age, diabetes and ischaemic heart disease.

## Data Availability

The data presented in this study are available on request from the corresponding author. The data are not publicly available due to privacy and confidentiality concerns.

## Appendix A. Strengthening the Reporting of Observational Studies in Epidemiology (STROBE)

This study was reported according to the Strengthening the Reporting of Observational Studies in Epidemiology (STROBE) checklist.

**Table A1:** STROBE statement—Checklist of items that should be included in reports of cohort studies.

	Item No	Recommendation	Page No
**Title and abstract**	1	(a) Indicate the study’s design with a commonly used term in the title or the abstract.	1
		(b) Provide in the abstract an informative and balanced summary of what was done and what was found.	1
**Introduction**			
Background/rationale	2	Explain the scientific background and rationale for the investigation being reported.	2
Objectives	3	State specific objectives, including any prespecified hypotheses.	3
**Methods**			
Study design	4	Present key elements of study design early in the paper.	3
Setting	5	Describe the setting, locations, and relevant dates, including periods of recruitment, exposure, follow-up, and data collection.	3, 4
Participants	6	(a) Give the eligibility criteria, and the sources and methods of selection of participants. Describe the methods of follow-up.	3, 4
		(b) For matched studies, provide the matching criteria and number of exposed and unexposed.	
Variables	7	Clearly define all the outcomes, exposures, predictors, potential confounders, and effect modifiers. Provide diagnostic criteria, if applicable.	4, 5
Data sources/measurement	8 *	For each variable of interest, provide the sources of data and details of the methods of assessment (measurement). Describe the comparability of the assessment methods if there is more than one group.	5
Bias	9	Describe any efforts to address potential sources of bias.	12
Study size	10	Explain how the study size was arrived at.	6
Quantitative variables	11	Explain how quantitative variables were handled in the analyses. If applicable, describe which groupings were chosen and why.	6
Statistical methods	12	(a) Describe all the statistical methods, including those used to control for confounding.	6, 7
		(b) Describe any methods used to examine subgroups and interactions.	6, 7
		(c) Explain how missing data were addressed.	N/A
		(d) If applicable, explain how loss to follow-up was addressed.	N/A
		(e) Describe any sensitivity analyses.	N/A
**Results**			
Participants	13 *	(a) Report numbers of individuals at each stage of study—e.g., numbers potentially eligible, examined for eligibility, confirmed eligible, included in the study, completing follow-up, and analysed.	8
		(b) provide reasons for non-participation at each stage.	
		(c) Consider use of a flow diagram.	
Descriptive data	14 *	(a) provide characteristics of study participants (e.g., demographic, clinical, social) and information on exposures and potential confounders.	8
		(b) Indicate number of participants with missing data for each variable of interest.	N/A
		(c) Summarise follow-up time (e.g., average and total amount).	9
Outcome data	15 *	Report numbers of outcome events or summary measures over time	9, 10
Main results	16	(a) Provide unadjusted estimates and, if applicable, confounder-adjusted estimates and their precision (e.g., 95% confidence interval). Make clear which confounders were adjusted for and why they were included.	10
		(b) Report category boundaries when continuous variables were categorised.	N/A
		(c) If relevant, consider translating estimates of relative risk into absolute risk for a meaningful time period.	N/A
Other analyses	17	Report other analyses performed—e.g., analyses of subgroups and interactions, and sensitivity analyses.	9, 10
**Discussion**			
Key results	18	Summarise key results with reference to study objectives.	11, 12
Limitations	19	Discuss limitations of the study, taking into account sources of potential bias or imprecision. Discuss both direction and magnitude of any potential bias.	12
Interpretation	20	Provide a cautious overall interpretation of results considering objectives, limitations, multiplicity of analyses, results from similar studies, and other relevant evidence.	12
Generalisability	21	Discuss the generalisability (external validity) of the study results.	12, 13
**Other information**			
Funding	22	Provide the source of funding and the role of the funders for the present study and, if applicable, for the original study on which the present article is based.	13

* Provide information separately for exposed and unexposed groups.

## Appendix B. Selection of the Cox Proportional Hazards Regression Model

Univariate Cox models were developed to determine the association between ATSI status and MALEs using risk factors that were either significantly different between the populations or those that were deemed to be clinically relevant in PAD. Multivariable regression models were developed by adjusting for the risk factors, including type 2 diabetes mellitus, age at recruitment, ischaemic heart disease (IHD), current smoking, and antithrombotic medications. Anti-glycaemic medication use was excluded from the model despite showing a significant difference between population groups to prevent multicollinearity with the inclusion of the type 2 diabetes mellitus variable. The best-fitting model was selected by calculating the Akaike information criterion (AIC) values for different models and performing a likelihood ratio test. The model performance of the best-fitting model was assessed using the concordance index. The outcomes of the best-fitting model were reported as the hazard ratio (HR) ± 95% confidence interval (CI). Additionally, the AIC values, likelihood ratio test, and concordance index for each model were compared (Table A2).

For MALEs, Model 1, which included just Aboriginal and Torres Strait Islander ethnicity, had the lowest AIC value (1671.991), and Models 2–6 all had delta-AIC values of less than 2, meaning they were not significantly different to Model 1 (Table A2). Model 7 had the highest AIC value and was thus deemed to be the worst-fit model. Of the models that included variables other than Aboriginal and Torres Strait Islander ethnicity, Model 4, including IHD, had the greatest concordance value (0.529). Since age and type 2 diabetes were found to be significantly different between the population groups, these risk factors were added to Model 4 to create Model 8. While the AIC value increased non-significantly, the concordance index improved compared to Model 4 (0.544 vs. 0.529). ANOVA testing showed that there was no significant difference between Model 8 and Model 1 (*p* = 0.4817). The Schoenfeld test was performed for Model 8 to assess the proportional hazard assumptions, and none of the *p* values were significant, suggesting that the model assumptions were not violated. Although there was no significant difference between Model 1 and Model 8 and the model assumptions were not violated, Model 8 was considered the parsimonious model as it addressed multiple known risk factors and had the highest concordance index.

For repeat revascularisation, Model 1, which included just Aboriginal and Torres Strait Islander ethnicity, had the lowest AIC value (1624.601), and Models 2–6 all had delta-AIC values of less than 2, meaning they were not significantly worse fits than Model 1 (Table A2). Inclusion of all the risk factors in Model 7 had the highest AIC value. Of the models that included variables other than Aboriginal and Torres Strait Islander ethnicity, Model 4, including IHD, had the greatest concordance value (0.538). Since age and type 2 diabetes were found to be significantly different between the population groups, these risk factors were added to Model 4 to create Model 8. While the AIC value increased non-significantly, the concordance index improved compared to Model 4 (0.546 vs. 0.538). ANOVA testing showed that there was no significant difference between Model 8 and Model 1 (*p* = 0.375). The Schoenfeld test was performed for Model 8 to assess the proportional hazard assumptions, and none of the *p* values were significant, suggesting that the model assumptions were not violated. Since there was no significant difference between Model 1 and Model 8 and the model assumptions were not violated, Model 8 was considered the parsimonious model as it addressed multiple known risk factors and had the highest concordance index.

**Table A2:** Comparison of the Cox proportional hazards regression models.

	Outcome: Major Adverse Limb Events					Outcome: Repeat Revascularisation			
Model Number	Predictor Variables	Hazard Ratio for Aboriginal and Torres Strait Islander Ethnicity (95% CI)	AIC Value	Likelihood Ratio Test	Concordance Index	Hazard Ratio for Aboriginal and Torres Strait Islander ethnicity (95% CI)	AIC Value	Likelihood Ratio Test	Concordance Index
1	- Aboriginal and Torres Strait Islander ethnicity	1.201 (0.6119–2.355)	1676.991	0.27	0.507	1.247 (0.6351–2.448)	1624.601	0.39	0.508
2	- Aboriginal and Torres Strait Islander ethnicity - Type 2 diabetes mellitus	1.133 (0.5676–2.262)	1678.468	0.79	0.519	1.165 (0.5831–2.329)	1625.899	1.09	0.524
3	- Aboriginal and Torres Strait Islander ethnicity - Age	1.1389 (0.5751–2.255)	1678.165	1.09	0.516	1.1847 (0.5976–2.348)	1625.854	1.13	0.517
4	- Aboriginal and Torres Strait Islander ethnicity - Ischaemic heart disease	1.159 (0.5893–2.281)	1677.780	1.48	0.529	1.206 (0.6124–2.374)	1625.544	1.44	0.538
5	- Aboriginal and Torres Strait Islander ethnicity - Current smoking	1.206 (0.6142–2.368)	1678.922	0.34	0.505	1.249 (0.6354–2.453)	1626.594	0.39	0.503
6	- Aboriginal and Torres Strait Islander ethnicity - Antithrombotic use	1.2264 (0.6242–2.409)	1677.980	1.28	0.508	1.2795 (0.6507–2.516)	1625.165	1.82	0.511
7	- Aboriginal and Torres Strait Islander ethnicity - Age - Type 2 diabetes mellitus - Ischaemic heart disease - Current smoking - Antithrombotic use	1.0396 (0.5110–2.115)	1682.493	4.77	0.54	1.0730 (0.5264–2.187)	1629.738	5.25	0.543
8	- Aboriginal and Torres Strait Islander ethnicity - Age - Diabetes - IHD	1.0162 (0.5016–2.059)	1679.954	3.31	0.544	1.0497 (0.5174–2.13)	1627.702	3.28	0.546

AIC = Akaike information criterion.
